# Percutaneous Ventricular Assist Device Fracture in the Right Ventricle and its Retrieval

**DOI:** 10.1016/j.jaccas.2022.04.026

**Published:** 2022-08-03

**Authors:** Faysal Alhasan, Hamza Rayes, Saavia Girgla, Divya Kompella, Mahboob Ali, Saad Ahmad

**Affiliations:** a Department of Internal Medicine, University of Cincinnati, Cincinnati, Ohio, USA; b Department of Cardiovascular Health and Disease, University of Cincinnati, Cincinnati, Ohio, USA; c Department of Cardiovascular Health and Disease, Beaumont Health Royal Oak, Royal Oak, Michigan, USA; d Department of Cardiovascular Health and Disease, Sutter Gould Medical Foundation, Tracy, California, USA

**Keywords:** complications, malfunction, mechanical support device, right heart failure, FDA, U.S. Food and Drug Administration, MAUDE, manufacturer and user facility device experience, PVAD, percutaneous ventricular assist device, RV, right ventricle, TTE, transthoracic echocardiogram

## Abstract

Mechanical circulatory support devices are used to offer short-term support for patients with cardiogenic shock. However, these devices are not without complications, and the risk and management of each must be closely considered. We discuss an infrequent complication of the percutaneous right heart pump and review complications reported to the U.S. Food and Drug Administration. (**Level of Difficulty: Intermediate.**)

## Central Illustration

**Figure:**
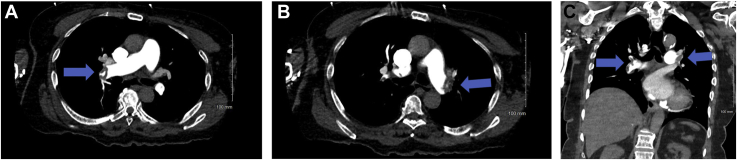

An 82-year-old woman presented from her skilled nursing home with a syncopal episode. She had been discharged 14 days earlier receiving aspirin 81 mg after hip replacement surgery. On arrival, she was alert but confused. She did not describe experiencing chest pain, shortness of breath, or palpitations. Her physical examination results were unremarkable, and her vital signs were notable for hypotension with blood pressure of 93/59 mm Hg, heart rate of 60 beats/min, respiratory rate of 18 breaths/min, and oxygen saturation of 87% on room air.

**Learning Objectives**

• To understand early detection of shock due to right ventricular failure in order to generate timely referrals for mechanical circulatory support.
• To recognize the importance of sheath structure, delivery, and durability during cardiac catheterization procedures.
• To understand the importance of appropriate tools and skill sets to be prepared for complications.
• To demonstrate cost-effective preparation and alternatives for any unforeseen procedural challenges, including having backup equipment available.

## Medical History

Her pertinent medical history included hypertension, for which she was taking metoprolol succinate 100 mg daily, type 2 diabetes mellitus, and chronic kidney disease stage 3.

## Differential Diagnosis

Given her presentation, our top differential diagnoses included cardiogenic shock in the setting of acute coronary syndrome, obstructive shock in the setting of massive pulmonary embolism, and distributive shock in the setting of infection.

## Investigations

**Figure 1:**
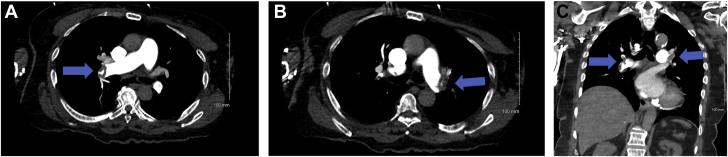
Bilateral Pulmonary Arteries Embolism **(A)** Right pulmonary artery emboli **(blue arrow)**, axial view. **(B)** Left pulmonary artery emboli **(blue arrow)**, axial view. **(C)** Bilateral pulmonary arteries emboli **(blue arrows)**, coronal view.

**Figure 2:**
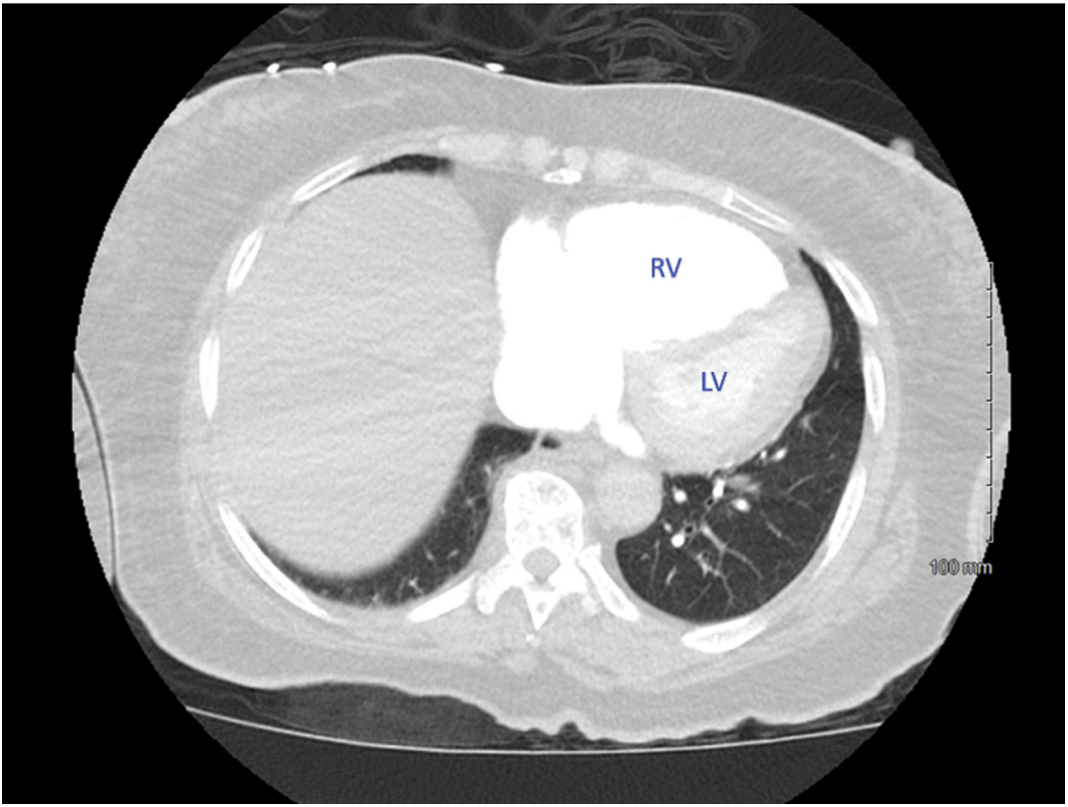
Dilation of Right Ventricle Dilated right ventricle (RV) on computed tomography scan. RV:left ventricle (LV) >1, consistent with RV strain.

An electrocardiogram showed sinus rhythm at 61 beats/min with normal axis and intervals, as well as new T-wave inversions in the anteroseptal leads with known T-wave inversions and Q waves in the inferoseptal leads. A limited transthoracic echocardiogram (TTE) showed normal left ventricular function and a dilated right ventricle (RV) with severely reduced function. Troponin was elevated at 3.39 ng/mL (reference range 0-0.03 ng/mL), and brain natriuretic peptide was notable at 2,013 pg/mL (reference range 0-100 pg/mL). Computed tomography of the chest showed large bilateral pulmonary emboli originating in the distal main pulmonary arteries and extending into almost all segmental branches (Figure 1) associated with right ventricular strain (Figure 2).

## Management

Given her recent hip surgery, the patient was not a candidate for systemic thrombolytic therapy. Therefore, we decided to treat her with catheter-directed thrombolytic agents. She underwent bilateral local alteplase infusion for 12 hours via each catheter at 1 mg/h. During this time, however, her shock worsened, and she required increasing epinephrine, norepinephrine, vasopressin, and dobutamine infusions, in addition to inhaled epoprostenol. Owing to refractory shock secondary to right-sided heart failure, the decision was made to escalate to mechanical circulatory support of the right ventricle.

**Figure 3:**
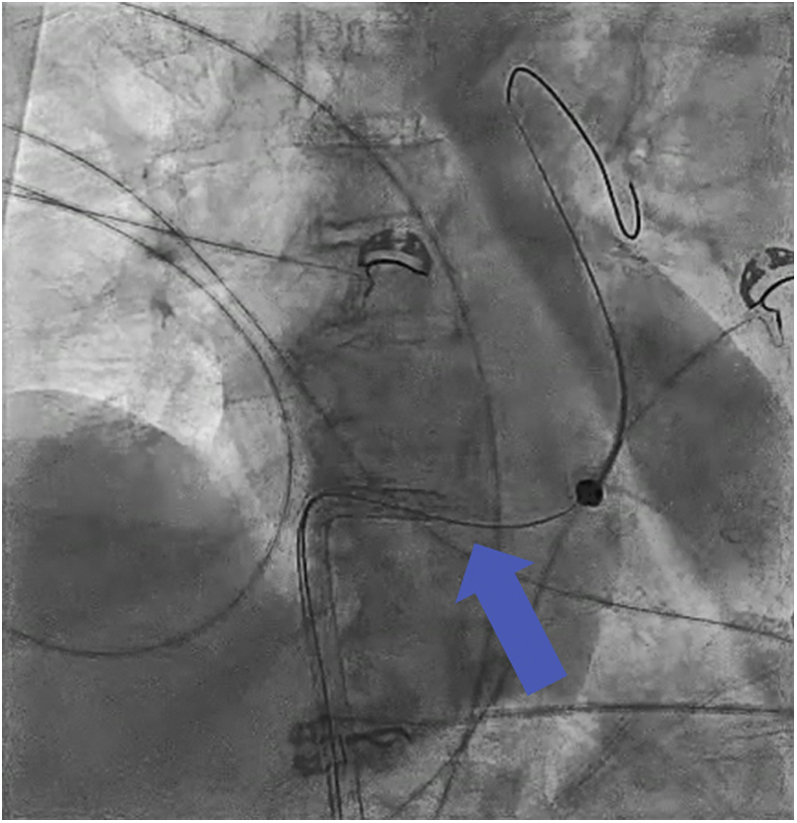
Separation of the Device Separated pigtail at the end of the device **(blue arrow)**.

**Figure 4:**
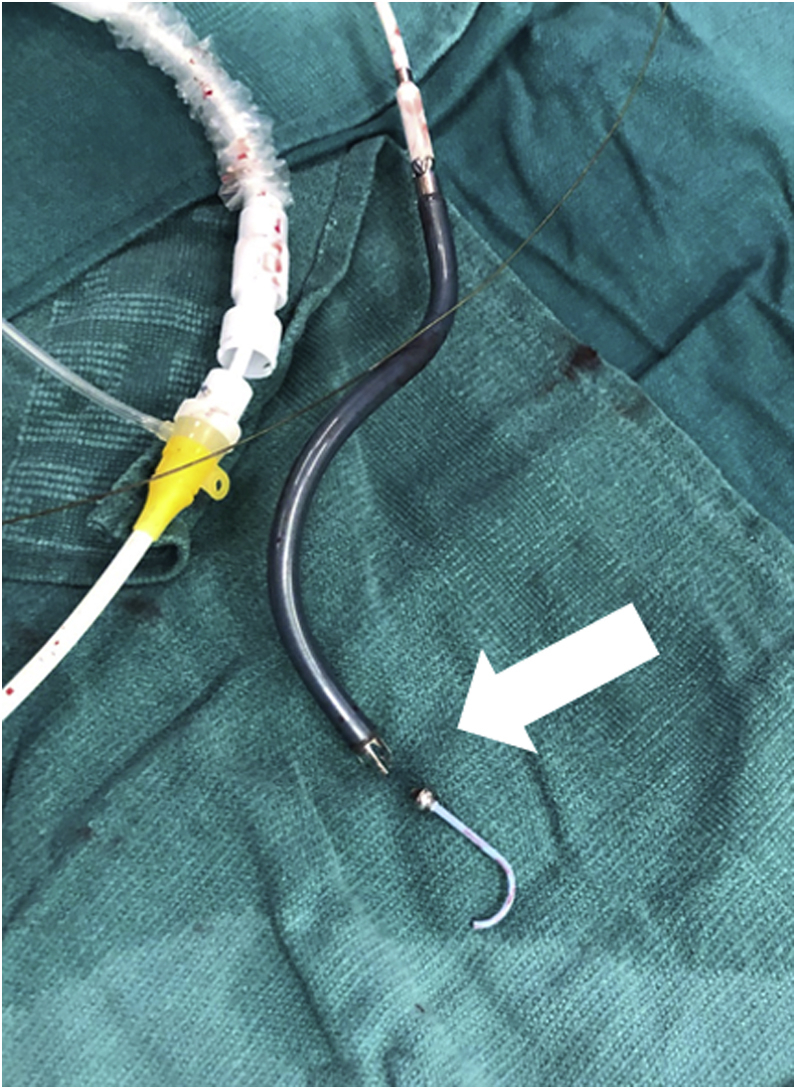
Direct Device Inspection Fracture between the device outlet and the teardrop metal piece **(white arrow)**.

**Figure 5:**
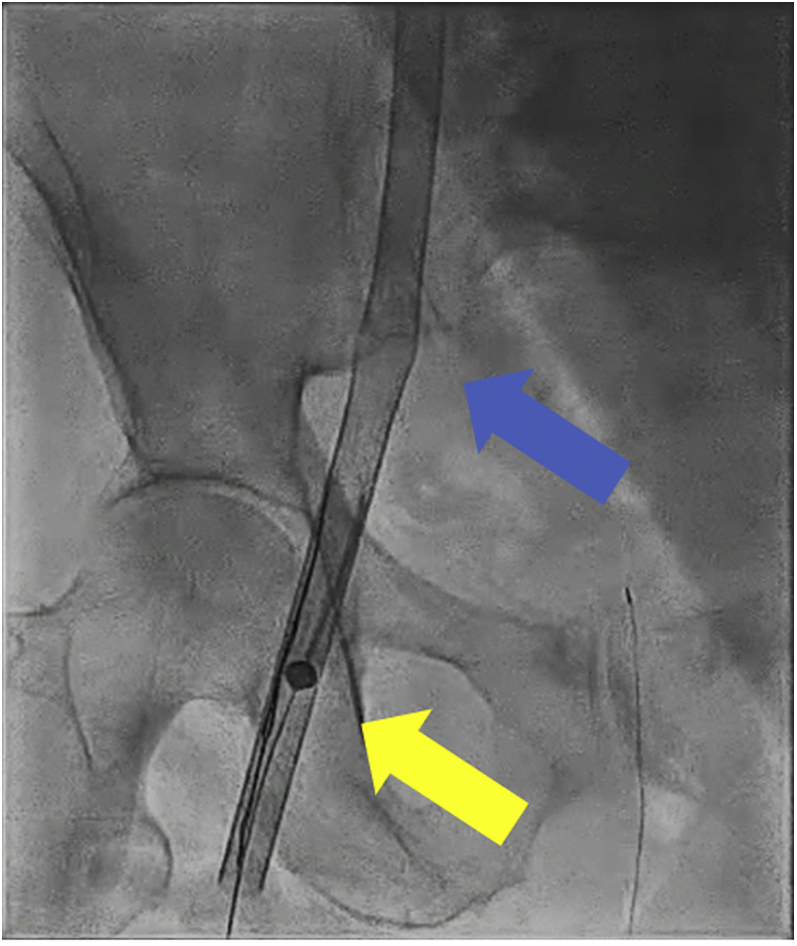
Fractured Device Capture Fractured device captured and retrieved successfully **(yellow arrow)**; sheath kink noted **(blue arrow)**.

**Figure 6:**
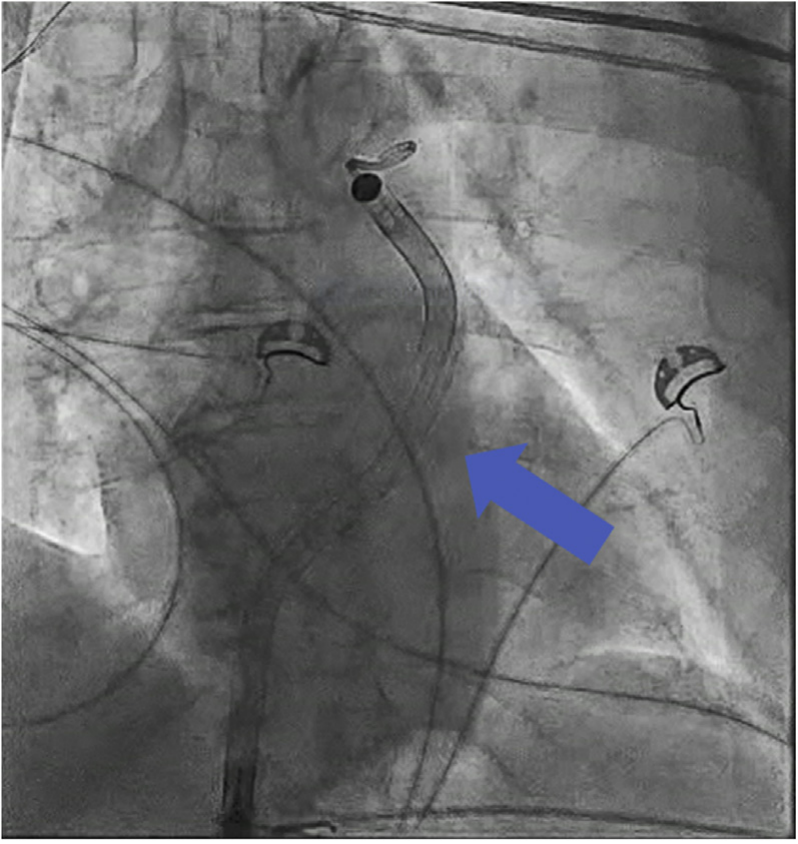
New Device Implant New device successfully implanted **(blue arrow)**.

The patient was taken to the cardiac catheterization laboratory, and the right femoral vein was accessed and upsized to the 23-F peel-away sheath after serial dilation. By use of the same access site, a stiff 0.018-inch wire was delivered into the left lower pulmonary artery via a Swan-Ganz catheter. An Impella RP device was then delivered over the wire. It was challenging to torque the device in the right ventricular outflow tract, and we observed that the outlet appeared deformed under fluoroscopy. Soon afterward, we witnessed separation of the pump at the outlet (Figure 3, Video 1). To retrieve the device, a 0.014-inch coronary work horse wire was advanced parallel to the fractured device into the right ventricular outflow tract. Next, an Ev3 15-mm Amplatz Goose Neck snare was advanced over it to successfully capture the distal end of the device wire. We then successfully pulled the wire and the device safely through the device sheath (Video 2). On direct inspection, we found a fracture between the outlet and the pigtail end (Figure 4). Additionally, a kink in the 23-F sheath was also noted (Figure 5, Video 3). We exchanged the kinked sheath for a new 23-F sheath, followed by an uneventful new device implantation (Figure 6). TTE after implantation showed a moderately dilated RV and moderately reduced RV systolic function (Video 4).

## Discussion

Percutaneously delivered right ventricular support devices are sophisticated devices approved by the U.S. Food and Drug Administration (FDA) for nonsurgical support of right ventricular failure.^1^ As with any cutting-edge technology, there are procedural challenges that must be considered. These challenges can be related to, but not limited to, patient comorbidities, operator expertise, or the device itself. In 2019, Khalid et al^2^ assessed the complications of the above-used right-sided percutaneous ventricular assist device (pVAD) by analyzing the postmarketing surveillance data from the FDA Manufacturer and User Facility Device Experience (MAUDE) database, which serves as a hub for voluntary reporting of complications. In the data queried for the period January 1, 2009, through December 31, 2018, the reported device-related complications included damage or fracture of device elements (34.2%), a thrombus in the system (17.1%), and device detachment (8.6%).

In our case, the device was inserted according to the manufacturer-directed instructions; however, the pigtail at the end of the device was found to be deformed (Figure 3).

Retrieval of a broken pigtail is procedurally complex and can have several implications. If not performed with care, the procedure can potentially cause damage to cardiac structures such as the tricuspid valve if the pigtail tip gets entangled within the chordae. Similarly, left-sided pVAD-related mitral valve injuries have also been reported.^3^ This risk can be significantly higher if the device tip is deformed or broken, because a hook-shaped tip would increase the likelihood that the device will become entangled with structures on its way out of the vascular system. In our case, we used the snare to manipulate and eventually retrieve the device safely. Direct inspection of the device showed that the device was broken between the tear drop and the outlet (Figure 4). Potential causes of such complications include manufacturing defects or deformation resulting from increased pressure during insertion. We believe that the kink in the sheath led to the deformation of the device upon insertion. In our experience, older device sheaths have shown susceptibility to kinking, which was also noted through the MAUDE database analysis.^2^ We also had instances when prepackaged left-sided pVAD long sheaths also became kinked, and an alternative manufacturer sheath was subsequently used successfully.

This event was reported to the FDA, and the device was returned to Abiomed for inspection. A result of the device investigation is pending.

## Follow-Up

We were able to wean the patient from epinephrine, norepinephrine, and vasopressin in the 8 hours after device implantation. The patient continued to use mechanical support for a total of 5 days, after which she proceeded to have a meaningful recovery. TTE 7 days after explantation of the device showed a mildly dilated RV but recovered RV systolic function (Video 5).

## Conclusions

Mechanical circulatory support devices are becoming essential tools in the management of refractory cardiogenic shock.^4^ The placement of such devices is associated with some procedural risk; however, it is critical to anticipate the complications related to the inherent device construct and its rare defects. Prompt recognition and early management of such challenges by an experienced operator are key in curtailing risks and improving outcomes.

## Funding Support and Author Disclosures

The authors have reported that they have no relationships relevant to the contents of this paper to disclose.

## Appendix

Supplemental Video 1 Separation of pigtail at the end of the device.

Supplemental Video 2 Deformed device being pulled into the sheath.

Supplemental Video 3 Fractured device capture and retrieval successfully; noted sheath kink.

Supplemental Video 4A Moderate RV dilation with reduced systolic function, TTE short axis view.

Supplemental Video 4B Moderate RV dilation with reduced systolic function, TTE apical view.5

Supplemental Video 4C Flattening of septum and D-shaped LV, TTE short axis view with contrast.6

Supplemental Video 5A Improved RV systolic function 7 days post device explant, TTE short axis view.7

Supplemental Video 5B Improved RV systolic function 7 days post device explant, TTE apical view.8
